# Gene Conversion Transfers the GAF-A Domain of Phosphodiesterase TbrPDEB1 to One Allele of TbrPDEB2 of *Trypanosoma brucei*

**DOI:** 10.1371/journal.pntd.0000455

**Published:** 2009-06-09

**Authors:** Stefan Kunz, Edith Luginbuehl, Thomas Seebeck

**Affiliations:** Institute of Cell Biology, University of Bern, Bern, Switzerland; New York University School of Medicine, United States of America

## Abstract

**Background:**

Chromosome 9 of *Trypanosoma brucei* contains two closely spaced, very similar open reading frames for cyclic nucleotide specific phosphodiesterases *TbrPDEB1* and *TbrPDEB2*. They are separated by 2379 bp, and both code for phosphodiesterases with two GAF domains in their N-terminal moieties and a catalytic domain at the C-terminus.

**Methods and Findings:**

The current study reveals that in the Lister427 strain of *T. brucei*, these two genes have undergone gene conversion, replacing the coding region for the GAF-A domain of *TbrPDEB2* by the corresponding region of the upstream gene *TbrPDEB1*. As a consequence, these strains express two slightly different versions of TbrPDEB2. TbrPDEB2a represents the wild-type phosphodiesterase, while TbrPDEB2b represents the product of the converted gene. Earlier work on the subcellular localization of TbrPDEB1 and TbrPDEB2 had demonstrated that TbrPDEB1 is predominantly located in the flagellum, whereas TbrPDEB2 partially locates to the flagellum but largely remains in the cell body. The current findings raised the question of whether this dual localization of TbrPDEB2 may reflect the two alleles. To resolve this, TbrPDEB2 of strain STIB247 that is homozygous for TbrPDEB2a was tagged in situ, and its intracellular localization was analyzed.

**Conclusions:**

The results obtained were very similar to those found earlier with Lister427, indicating that the dual localization of TbrPDEB2 reflects its true function and is not simply due to the presence of the two different alleles. Notably, the gene conversion event is unique for the Lister427 strain and all its derivatives. Based on this finding, a convenient PCR test has been developed that allows the stringent discrimination between Lister-derived strains that are common in many laboratories and other isolates. The technique is likely very useful to resolve questions about potential mix-ups of precious field isolates with the ubiquitous Lister strain.

## Author Summary

Cyclic nucleotide specific phosphodiesterases are important regulators of cyclic nucleotide signalling in eukaryotes. In many organisms, including humans and trypanosomes, some of these enzymes contain specific domains (GAF domains) that bind cyclic nucleotides, and that are involved in the regulation of the catalytic domain. In the parasitic protozoon that causes human sleeping sickness, *Trypanosoma brucei*, two closely related phosphodiesterases each contain two such GAF domains, GAF-A and GAF-B. Their genes are tandemly located on chromosome 9, spaced by only a few thousand nucleotides. We here show that a gene conversion event has exchanged the region that codes for the GAF-A domain of the downstream gene by the closely similar corresponding sequence of the upstream gene. This domain exchange has no effect on intracellular localization of the two enzymes. The gene conversion event has occurred in one particular strain of trypanosomes (Lister427) and is found in all its derivatives, but not in any other strain or isolate. The presence or absence of this gene conversion represents a useful analytical marker for the stringent discrimination of Lister427 derivatives from other trypanosome strains.

## Introduction

Cyclic nucleotide-specific phosphodiesterases (PDEs) are crucial players in cyclic nucleotide signalling of eukaryotic cells. Several of the human PDEs have become important drug targets, and all eleven human PDE families are under intense study for the development of new therapeutic PDE-inhibitors [1]. Over the last few years, the PDEs of protozoal parasites have also come into focus as potential targets for new and effective antiparasitic drugs [2]–[4]. The genomes of all kinetoplastid parasites, including *Trypanosoma brucei*, contain similar sets of genes that code for cyclic nucleotide-specific phosphodiesterases (PDEs; [2],[5]). Three of these, *PDEA* (Tb10.389.0510), *PDEC* (Tb03.27C5.640) and *PDED* (Tb03.3K10.420) are single copy genes located on different chromosomes, while two closely similar genes that code for the cAMP-specific phosphodiesterases PDEB1 and PDEB2 are tandemly clustered. In *T. brucei*, these two genes, *TbrPDEB1* (Tb09.160.3590) and *TbrPDEB2* (Tb09.160.3630), are located on chromosome 9, and their open reading frames are separated by 2379 bp. They code for very similar proteins of 930 and 925 amino acids, respectively. Both consist of a less-conserved N-terminal region of about 200 amino acids (44.8% amino acid sequence identity between TbrPDEB1 and TbrPDEB2), followed by two highly conserved GAF domains [6], GAF-A (94.4% identity) and GAF-B (100% identity) and a catalytic domain (90.8% identity) [2]. The GAF-A domains of both proteins bind cAMP (TbrPDEB1: A. Schmid, unpublished; TbrPDEB2: [7]) and might function as allosteric regulators of enzyme activity. The precise function and potential ligand specificity of the GAF-B domains are currently unknown. Based on structural analyses with human PDEs that contain GAF domains [8], they might be involved in dimer formation. Despite the extensive overall sequence conservation between PDEs TbrPDEB1 and TbrPDEB2, their subcellular localization is distinct. TbrPDEB1 is located predominantly in the flagellum, with which it remains tightly associated after detergent extraction of the cells. In contrast, TbrPDEB2 is mainly located in the cytoplasm as a soluble enzyme, with only a small proportion also locating to the flagellum [3]. Considering the relatively low degree of sequence conservation between the N-terminal regions of TbrPDEB1 and TbrPDEB2, these regions, and/or the GAF-A domains might contain the signals for intracellular localization.

This study reports the occurrence of a gene conversion event between the two tandemly arranged genes *TbrPDEB1* and *TbrPDEB2*. Gene conversion in *T. brucei* has so far been mainly studied in the context of variable surface proteins [9]–[11], where it is the predominant, though not the only mechanism that drives antigenic variation [12]. Homologous recombination and gene conversion are fundamental processes of genome biology that are involved in a broad range of cellular functions including DNA repair, telomere maintenance, DNA replication and meiotic chromosome segregation [13]. Thus, one might safely assume that they play similarly important roles in trypanosomes and are not restricted to the realm of antigenic variation. Depending on organism and cell division mode, the length of gene-conversion tracts varies considerably. In the yeast *S. cerevisiae*, mitotic gene conversion tracts are often larger than 4 kb, while the meiotic tracts are usually between 1 and 2 kb. In mammals, on the other hand, mitotic gene conversion tracts are usually between 200 and 1000 bp in length [13]. In *T. brucei*, gene conversion tracts of variable surface glycoprotein genes are usually around 3 kb [9]. Despite a long history of studying antigenic variation [14]–[18], our understanding of the precise role and the mechanistic details of gene conversion in antigenic variation is still limited. Even less is known about these processes in regions of the genome that are not involved in antigenic variation. Recent data have shown that the efficiency of homologous recombination depends on target length and sequence conservation [19] and suggests that two distinct recombination mechanisms might be active in trypanosomes. Interestingly, *T. brucei* BRCA2, a prominent player in homologous recombination, has acquired an unusually high number (twelve) of BRC repeats within its N-terminal domain [11].

The current study describes the occurrence of a gene conversion of several hundred bp within the coding region of the *TbrPDEB2* gene by the corresponding region of the *TbrPDEB1* gene. The gene conversion does not affect the intracellular localization of the TbrPDEB2 gene product. This event is unique for the Lister strain of *T. brucei* [20] and all its derivatives, but it is not found in other *T. brucei* strains. The presence of this particular gene conversion serves as a useful genetic marker to discriminate Lister derivatives from other *T. brucei* strains.

## Methods

### Trypanosome culture

Procyclic trypanosomes were cultured in SDM-79 medium containing 5% FCS [21], and bloodstream forms were grown in HMI-9 medium containing 10% FCS [22]. The following strains were used: the procyclic strain Lister427 [23], the bloodstream form of Lister 427-2 (strain 221; MiTat1.2; [24]), STIB247, STIB345AD (a derivative of EATRO1529, which was isolated from *Glossina pallipides* in Kiboko, Kenya in 1969 and cryopreserved after six passages in mice. In 1973, it was stabilated after five short passages in rats and renamed STIB345), GVR35 (isolated 1966 in the Serengeti), AnTat 1.1 [25], and the 427-derived SM strain [26]. Genomic DNAs of strains 427 variant 3 and TREU927 [27] were generously supplied by Wendy Gibson (University of Bristol, UK), and genomic DNA of the *T. b. rhodesiense* strain STIB900 was a gift of Barbara Nerima (University of Bern). STIB900 was isolated as STIB704 in Ifakara, Tanzania in 1981 from a male patient. It was cloned and adapted to axenic culture. A detailed pedigree of many trypanosome isolates and derivatives can be found at http://tryps.rockefeller.edu/trypsru2_pedigrees.html as well as in a recent paper [20].

### PCR primers

The following primers were used for PCR:

TbrB2-for (28-mer; 53.6% GC, T_m_ 63°C, specific for TbrPDEB2): C_556_ACGCCTCTACGATGCTTGAGTCATCAC

TbrB1-for (26-mer, 57.7% GC, T_m_ 63°C, specific for TbrPDEB1; used for duplex PCR)

G_258_ATGGAGCACACAATGACGCACGGTG

TbrGAFA1-rev (29-mer, 51.7% GC, T_m_ 63°C, specific for converted allele TbrPDEB2b, nucleotides 854–827)

C_854_CTACAATGCCTGTTCCCTTGGGTATGGA

TbrGAFA2-rev (27-mer, 55.6% GC, T_m_ 63°C, specific for wild-type allele TbrPDEB2a, nucleotides 852–827)

G_852_GCAATACCTGCACCCCTAGGGATTGT

### PCR amplification

As a template, genomic DNA was used in all reactions (100 ng/reaction). The annealing temperature of the primers was optimized using gradient PCR, and the final cycling protocol was as follows: an initial denaturation step of 4 min at 94°C, followed by 31 cycles consisting of 1 min at 94°C, 1 min at 63°C and 1 min at 72°C, followed by a final extension step of 10 min at 72°C. Typical reactions (20 µl) with single primer pairs contained 100 ng genomic DNA, 300 nM of each primer, 500 µM of each NTP and 1 unit Taq polymerase. Reactions for duplex PCR contained 300 nM each of primers TbrB1-for and TbrB2-for, and 400 nM TbrGAFA1-rev.

### In situ tagging of TbrPDEB2 in procyclic STIB247

Procyclic forms of strain STIB247 as a representative strain that is homozygous for TbrPDEB2, i.e. has not undergone gene conversion of one allele, were used to explore the intracellular localization of TbrPDEB2. The cells were transfected with a construct that introduces a C-terminal triple c-Myc tag into one copy of TbrPDEB2 [3],[28]. Transformation and selection of clones were done exactly as recently described [29]. Transfectants were selected with 25 µg/ml hygromycin, cloned and verified by Southern blotting, and protein expression was confirmed by Western blotting. Four independent clones were then used for immunofluorescence microscopy.

### Immunofluorescence microscopy

Immunofluorescence microscopy was done as described previously [3]. Cells were fixed with 4% PFA in PBS and permeabilized with methanol for 10 min at −20°C. For the preparation of cytoskeletons, cells were extracted once with cold MME buffer (100 mM HEPES, pH 6.9, 1 mM MgSO_4_, 1 mM EGTA) containing 0.5% Triton X-100 for 5 min, prior to the fixation with 4% PFA. The first antibody was a monoclonal mouse anti-c-Myc antibody (9E10, Santa Cruz) diluted 1∶200 in PBS+2.5% BSA (w/v). The secondary antibody was Alexa Fluor 488 conjugated goat anti-mouse polyclonal antibody (Molecular Probes) diluted 1∶750. Coverslips were mounted with Vectashield mounting medium containing DAPI (Vector Laboratories), and slides were analyzed with a Leica DM6000B microscope.

### TX-100 fractionation

About 1×10^7^ trypanosomes were washed once in PBS and then lysed on ice for 10 min in PBS/0.5% Triton X-100, supplemented with protease inhibitor (Roche Complete Mini, EDTA-free). The extracted cells were centrifuged for 10 min at 13,000 rpm at 4°C. Supernatants and pellets were analyzed by Western blotting. Gels were transferred to nitrocellulose filters and probed with mouse anti c-Myc 9E10 antibody (Santa Cruz; diluted 1∶1000). Control antibodies were polyclonal rabbit anti BiP (endoplasmic reticulum staining; as a marker for Triton soluble proteins; 1∶50,000; gift of Jay Bangs, University of Wisconsin, Madison) and polyclonal rat anti PFR (as a marker for Triton insoluble proteins; 1∶30,000).

## Results

**Figure 1:**
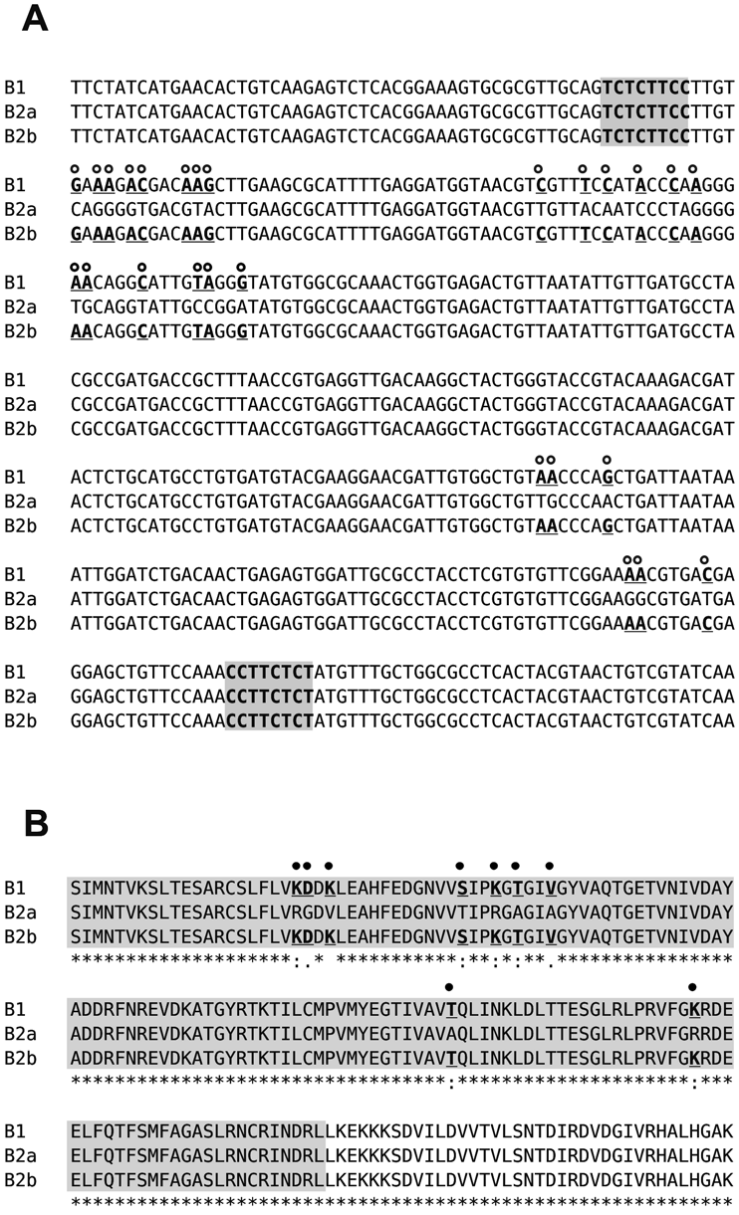
Gene conversion between TbrPDEB1 and TbrPDEB2 in the region coding for the GAF-A domain. Panel A: B1: DNA sequence of TbrPDEB1 (nucleotides 720–1139 of the open reading frame of TbrPDEB1); B2a: DNA sequence of the wild-type allele of TbrPDEB2; B2b: DNA sequence of the allele having undergone gene conversion with TbrPDEB1. Open circles, bold, underlined: nucleotide changes due to the conversion event; shaded: polypyrimidine stretches before and after converted segment. Panel B: corresponding amino acid sequences (amino acids 241–420 of TbrPDEB1). Closed circles, bold, underlined: amino acid substitutions due to the conversion event; shaded: GAF-A domain.

The genome of *T. brucei* contains two tandemly arranged open reading frames for the phosphodiesterases TbrPDEB1 and TbrPDEB2. They are located on chromosome 9 and are separated by 2379 base pairs [3],[30]. Sequencing of the two genes from our standard laboratory strain Lister427 unexpectedly revealed the presence of two slightly distinct versions of the *TbrPDEB2* gene in the genome of this strain (Fig. 1). One allele corresponded to that present in the *T. brucei* database (*TbrPDEB2a*). However, in the second allele (*TbrPDEB2b*), a part of the sequence that codes for the GAF-A domain was replaced by the corresponding sequence from *TbrPDEB*1 (Fig. 1). Since *TbrPDEB1* and *TbrPDEB2* are tandemly arranged and closely spaced (see above), ectopic gene conversion between the two is a likely scenario, though interchromatid or interchromosomal gene conversion cannot be excluded. The length of the minimal converted tract is 297 bp (n 780–1077 of both genes), while its maximal length [13] is 660 bp (n 702–1362). The length of the converted tract is much shorter than the length of tracts found in mitotic gene conversion in *S. cerevisiae* [31], but it is in good agreement with what is found in mammals [32],[33]. DNA sequence conservation between the corresponding regions of *TbrPDEB1* and *TbrPDEB2* is high (91.5 and 96.1% for the minimal and maximal converted tracts, respectively), making them a plausible template for gene conversion [13]. The conversion results in a change of twenty-six base-pairs in the sequence of *TbrPDEB2b*. These changes translate into nine amino acid substitutions, of which seven are conservative (R_261_K, T_276_S, R_279_K, A_281_T, A_284_V, A_335_T, and R_357_K) and two are not (G_262_D and V_264_K).

Interestingly, the putative minimal converted tract is flanked by two short pyrimidine-stretches (T_767_CTCTTCCTT_776_ upstream, and C_1094_CTTCTCT_1101_ downstream) that might facilitate the onset of gene conversion [13].

**Figure 2:**
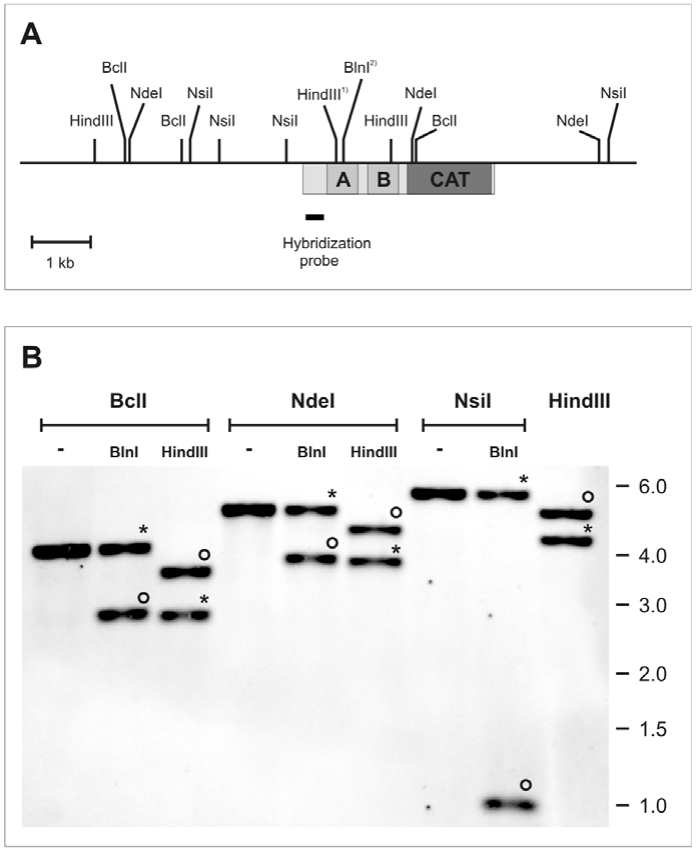
Restriction enzyme analysis of TbrPDEB2a and TbrPDEB2b. Panel A: Restriction map of the TbrPDEB2 locus on chromosome 9. HindIII^1)^: site present only in the converted allele TbrPDEB2b; BlnI^2)^: site present only in non-converted allele TbrPDEB2a. Box underneath horizontal line: Open reading frame of TbrPDEB2. A,B: GAF domains, CAT: catalytic domain. Hybridization probe: detects both alleles, TbrPDEB2a and TbrPDEB2b; corresponds to nucleotides 158–518 of TbrPDEB2. Panel B: Southern blot analysis of singly or doubly digested genomic DNA establishes the presence of both alleles in the genome of strain Lister427. Circles: Fragments derived from allele TbrPDEB2a; asterisks: fragments derived from allele TbrPDEB2b; no tag: fragments common to both. Sizes of molecular weight markers are indicated to the right.

To exclude the possibility of PCR and/or sequencing artefacts, the presence of the two alleles in the genome was further established by restriction enzyme analysis and Southern blot hybridization (Fig. 2). The restriction enzyme BclI cuts once in the upstream region and once within the open reading frame of both alleles of *TbrPDEB2*, resulting in a single band of 4.2 kbp when hybridized with a probe that is specific for the common 5′-terminal region (nucleotides 158–518). Allele *TbrPDEB2a* contains a unique BlnI site (bp 832–837) that is abolished in the converted allele *TbrPDEB2b*. As expected, a double digest of genomic DNA with BclI and BlnI, followed by hybridization with the 5′-specific probe produces two bands. The band at 4.4 kbp corresponds to allele *TbrPDEB2b* that has lost its BlnI site, while the band at 2.8 kbp represents allele *TbrPDEB2a*. The two alleles can be further discriminated by HindIII digestion. HindIII has a common cutting site in both (bp 1552–1556). An additional HindIII site is only present in the converted DNA stretch of *TbrPDE2b* (bp 790–794). When genomic DNA is digested with BclI and HindIII and hybridized as above, two bands are again detected. The upper band (3.6 kbp) corresponds to *TbrPDEB2a*, while the lower band (2.8 kbp) reflects the additional HindIII site in *TbrPDE2b*. The analogous reasoning was followed when using double digestions BlnI or HindIII in combination with NdeI, or with BlnI or HindIII in combination with NsiI (Fig. 2B). All blots were also hybridized with a *TbrPDEB1*-specific probe to make sure that the *TbrPDEB1* gene has remained unaltered. The digests are in good agreement with the predictions and confirm the presence of two distinct *TbrPDEB2* alleles in the genome. In conjunction, these results demonstrate that one allele of *TbrPDEB2* has undergone ectopic gene conversion with the *TbrPDEB1* gene, with the effect of replacing the stretch of DNA sequence that codes for the GAF-A domain with the corresponding region of the *TbrPDEB1* gene.

### Gene conversion is specific for the Lister427 strain and its derivatives

**Figure 3:**
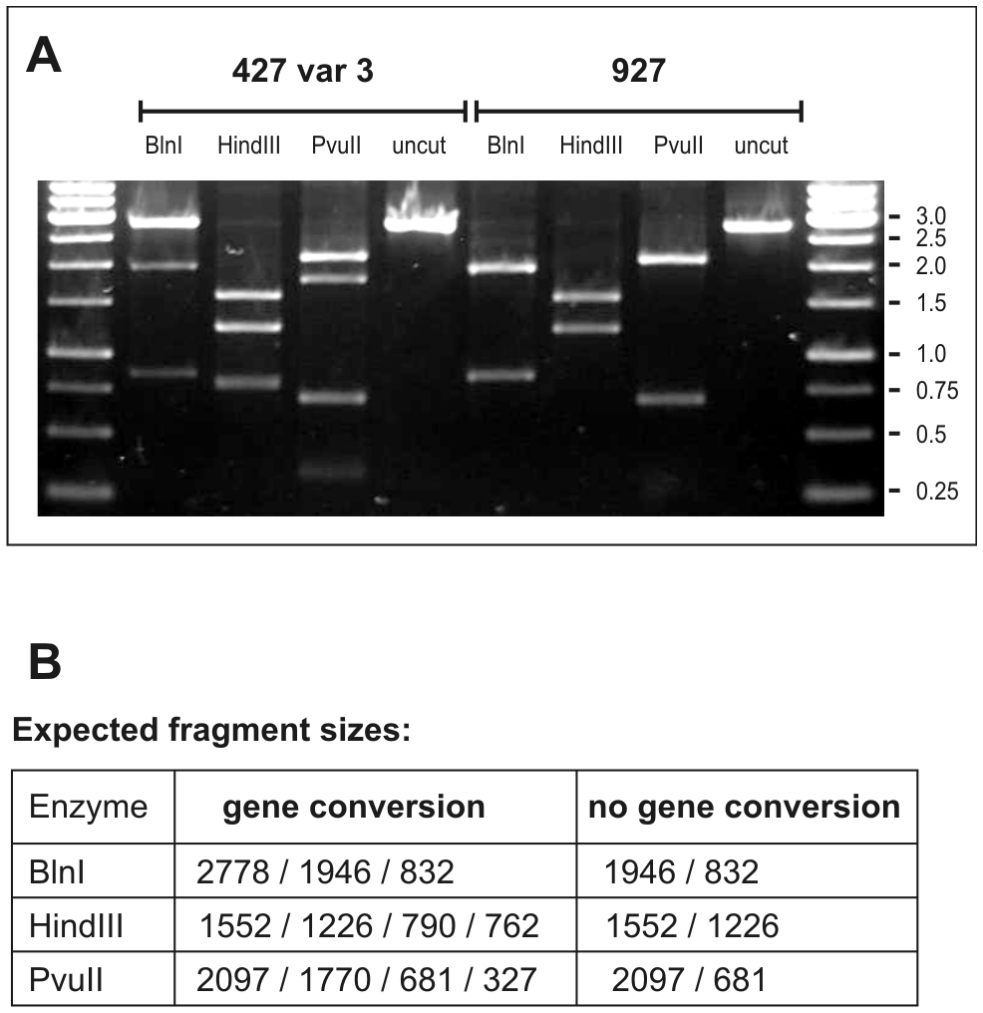
Restriction enzyme analysis of the wild-type and converted TbrPDEB2 open reading frames. These were amplified from genomic DNA of strain 427var3 (containing one converted allele) and strain 927 (no gene conversion). Panel A: restriction digests. Panel B: expected fragment sizes correspond exactly to those obtained experimentally.

In an effort to determine if the observed gene conversion represents an ancient event of the history of *T. brucei*, and thus can be found in many independent isolates, the genomic DNA of a number of independent strains with different histories were analyzed. The entire open reading frame of TbrPDEB2 was PCR amplified, followed by restriction enzyme analysis of the PCR products. As demonstrated in Fig. 3, this procedure allowed the unambiguous discrimination between strains that had undergone the gene conversion event and those that did not (Table 1). The occurrence of gene conversion was initially detected in the procyclic strain 427, our standard laboratory strain. This strain most likely is a derivative of the Lister427 strain (http://tryps.rockefeller.edu/trypsru2_pedigrees.html), which in turn is probably derived from the Shinayaga III strain isolated in 1956 from cattle in Tanganyika. The other two Lister427 derivatives that were analyzed (BS221, the “NewYork single marker” strain derived in 1999 from BS221 [26], and the ancestral 427 variant 3 strain documented and frozen down in 1964 at the Lister Institute) were also heterozygous at the TbrPDEB2 locus, containing one allele for TbrPDEB2a and one for TbrPDEB2b. This indicated that the conversion, once it had occurred, was stably maintained. In contrast, a number of other strains and isolates (AnTat1.1 (isolated 1966 in Uganda), GVR35 (isolated 1966 in the Serengeti), STIB247 (isolated in 1971 in the Serengeti), STIB345AD (isolated in 1969 in Kenya), TREU927 [27] and the *T. b. rhodesiense* strain STIB900) had not undergone gene conversion at this locus.

**Table 1:** Listing of strains, their history and TbrPDEB2 genotype.

Strain	Ancestry	Gene conversion
Procyclic 427	Lister427	Yes
MiTat1.2(221)	Lister427	Yes
NYSM	Lister427	Yes
427 var 3	Lister427	Yes
GVR35	Serengeti 1966	No
STIB247	Serengeti 1971	No
STIB354AD	Kiboko, Kenya 1969	No
TREU927	Kiboko, Kenya 1969/70	No
TREU667	East Africa, ca. 1965	No
AnTat1.1	Uganda 1966	No
STIB900	Ifakara, Tanzania, 1981 (*T.b. rhodesiense*)	No

### Gene conversion as a convenient identifier of 427-derived strains

**Figure 4:**
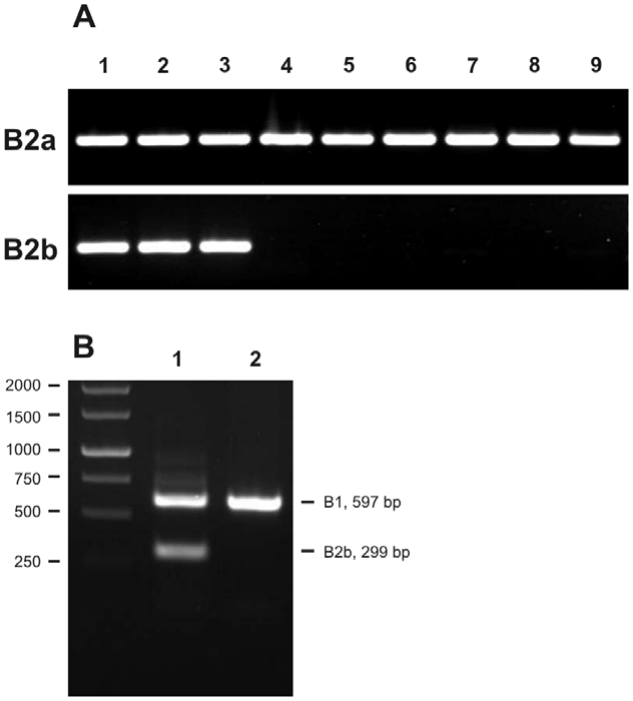
Strain discrimination based on the presence or absence of gene conversion in TbrPDEB2. A: upper panel: amplification with primer pair TbrB2-for and TbrGAFA2-rev (specific for the wild-type allele); lower panel: amplification with primer pair TbrB2-for and TbrGAFA1-rev (specific for the converted allele). Templates were genomic DNAs of the following strains: 1: procyclic 427; 2: BS221; 3: 427var3; 4: 927; 5: AnTat1.1; 6: STIB247; 7: GVR 35; 8: STIB345; 9: STIB900 (*T. b. rhodesiense*). B: Duplex PCR for the simultaneous detection of TbrPDEB1 (as an internal control) and the converted allele TbrPDEB2b, using the three primers TbrB1-for, TbrB2-for and TbrGAFA1-rev. Templates were genomic DNAs of strains 427 (1) and STIB247 (2). The 597 bp fragment from TbrPDEB1 corresponds to two gene equivalents, while the 299 bp fragment from TbrPDEB2b corresponds to one gene equivalent.

In the past, the precise origin of trypanosomal strains has often caused much debate. In particular, accidental mixups between the ubiquitous laboratory strains of the 427 lineage and other strains have remained a lingering concern (e.g. see http://tryps.rockefeller.edu/trypsru2_pedigrees.html). The presence of the gene conversion in *TbrPDEB2* of the 427 lineage now provides a rapid and convenient means of strain identification. The presence or absence of the gene conversion can be monitored by using two sets of specific primers (Fig. 4). The primer set that is specific for the unconverted GAF-A region of *TbrPDEB2a* (TbrB2-for and TbrGAFA2-rev) produces an amplicon of 297 bp from genomic DNA of every *T. brucei* strain tested. In contrast, the primer set with specificity for the converted gene *TbrPDEB2b* (TbrB2-for and TbrGAFA1-rev) produces a PCR product (299 bp) only with genomic DNA from 427 derivative strains. Thus, these two primer sets represent a convenient diagnostic tool for strain identification in cases where contamination or confusion of a strain with a 427 derivative is suspected. A duplex PCR setup using a combination of the three primers TbrB1-for, TbrB2for and TbrGAFA1-rev (see Methods) allows the simultaneous detection of the presence of TbrPDEB1 (as a positive control) and the presence or absence of the TbrPDEB2b allele (Fig. 4B).

### Intracellular localization of TbrPDEB2 is unaltered in a strain that has not undergone gene conversion

**Figure 5:**
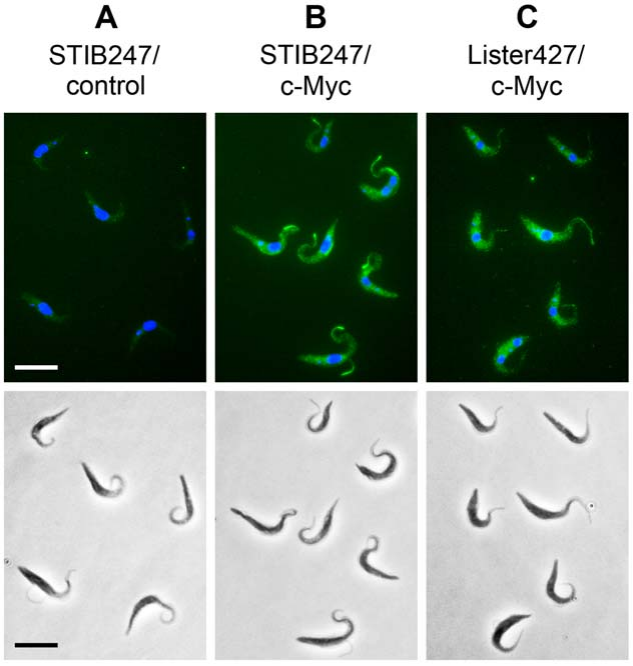
Subcellular localization of TbrPDEB2 is not altered by gene conversion. Immunoflourescence analysis of c-Myc tagged TbrPDEB2 alleles. Panel A: STIB247 wt (negative control); panel B: STIB247, homozygous for wild-type TbrPDEB2a, one gene copy C-terminally tagged with c-Myc; panel C: Lister 427, heterozygous; the gene-converted allele TbrPDEB2b is tagged with c-Myc. Upper row: Alexa Fluor 488 and DAPI fluorescence; bottom row: phase contrast microscopy. Bars represent 10 um.

Earlier work from this laboratory using strain 427 has demonstrated that TbrPDEB2 is located partly in the cytoplasm and partly in the flagellum [3]. A recent analysis demonstrated that in the strain used for these experiments, the tag had been integrated into the TbrPDEB2b allele. This raised the question if the observed intracellular localization was specific for the B2b allele, and might be different from the localization of the B2a gene product. The question was all the more pertinent as in many mammalian PDEs, the information for intracellular localization is contained in the N-terminal part of the PDE molecules [8]. To answer this question, one allele of the TbrPDEB2 gene of a strain that has not undergone gene conversion (procyclic STIB247, see above) was tagged in-situ with a 3× c-Myc tag [3],[28]. Expression of the protein and its intracellular localization were analyzed and compared with the earlier results obtained with the 427 strain (Fig. 5). Though cell shape and motility are rather different between strain Lister427 and STIB247, no major differences in intracellular localization of the tagged TbrPDEB2 could be detected between the strains 427 (containing the gene conversion) and STIB247 (no gene conversion). In a similar experiment with bloodstream forms of BS221, either TbrPDEB2a or TbrPDEB2b were tagged with a c-Myc tag. Both gene products showed an identical subcellular localization. In conjunction, these observations indicate that the slight alterations in the amino acid sequence of the GAF-A domain of TbrPDEB2b do not affect the intracellular localization of the enzyme. Furthermore, they demonstrate that the presence of TbrPDEB2 both in the cell body and in the flagellum is not simply due to the presence of two slightly distinct alleles, but rather reflects a genuine property of the enzyme.

**Figure 6:**
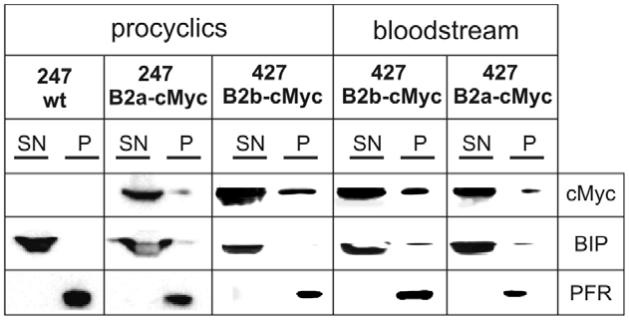
Triton X-100 solubility of TbrPDEB2 is not altered by gene conversion. Immunoblots of Triton X-100 soluble (SN) and insoluble (P) fractions developped with antibodies against c-Myc, BiP and PFR. Strains analyzed: 247wt: negative control (no c-Myc tagged protein); 247 cMyc: wild-type allele TbrPDEB2a tagged with c-Myc; 427 cMyc: gene-converted allele TbrPDEB2b tagged with c-Myc; B2b-cMyc: heterozygous strain Lister427, gene-converted allele TbrPDEB2b tagged with c-Myc; B2a-cMyc: heterozygous strain Lister427, wild-type allele TbrPDEB2a tagged with c-Myc.

**Figure 7:**
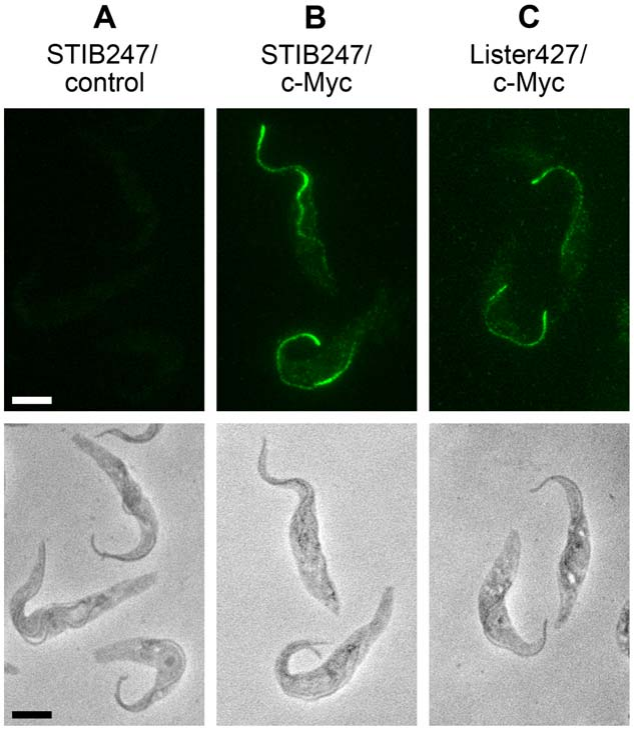
Immunofluorescence of Triton-X100 extracted cytoskeletons of procyclic trypanosomes. Triton-resistant, c-Myc tagged TbrPDEB2a and B2b (see Fig. 6) both remain associated with the flagellum. Panel A: STIB247 wt; panel B: STIB427 with tagged TbrPDEB2a; panel C: 427 with tagged TbrPDEB2b.

The distribution of TbrPDEB2a and TbrPDEB2b were further analyzed using Triton X-100 solubilization. Earlier work had shown that TbrPDEB2 is mostly Triton soluble, with a minor fraction remaining Triton-insoluble [3]. To determine if the gene conversion might alter the Triton solubility of the affected TbrPDEB2 allele, the following cell lines were fractionated with Triton X-100: procyclic 247 wild type (negative control), procyclic 247 (homozygous for TbrPDEB2a, one allele tagged with c-Myc), procyclic 427 (gene-converted TbrPDEB2b allele tagged with c-Myc), bloodstream form Lister427 (gene-converted TbrPDEB2b allele tagged with c-Myc) and bloodstream form Lister427 (wild-type TbrPDEB2a allele tagged with c-Myc) (Fig. 6). The blots were probed with anti c-Myc antibody, and subsequently with antibodies against BiP (fully Triton soluble; [34]) and PFR (fully Triton insoluble; [35]) as controls. In all strains, the TbrPDEB2 alleles behaved identically, the majority of the protein being Triton-soluble, with a minor portion remaining in the insoluble pellet. Triton extracted cytoskeletons were also analyzed by immunofluorescence (Fig. 7). Triton X-100 resistant TbrPDEB2a and TbrPDEB2b both localize along the flagellum. In conjunction, these data demonstrate that the gene conversion between TbrPDEB1 and one allele of TbrPDEB2 does not alter the subcellular localization of the gene product TbrPDEB2b that is produced from the converted allele.

## Discussion

The current study describes a gene conversion event that has occurred between the GAF domains of the tandemly arranged genes for phosphodiesterases TbrPDEB1 and TbrPDEB2 on chromosome 9 of *T. brucei*. It demonstrates that this gene conversion does not affect the subcellular localization of the gene product TbrPDEB2b. In addition, the study demonstrates that this gene conversion provides a sensitive marker to discriminate Lister427 derivatives from other trypanosome strains. In the *T. brucei* strain Lister427, one allele of the *TbrPDEB2* gene has undergone a gene conversion which replaces a stretch of the gene with the corresponding region of the upstream gene *TbrPDEB1*. The tandemly arranged open reading frames of *TbrPDEB1* and *TbrPDEB2* are separated by a mere 2379 bp, and they share a high degree of sequence identity, in particular between the regions that code for the two GAF domains and for the catalytic domain. The conversion generated two non-identical alleles of *TbrPDEB2*, *TbrPDEB2a* (no gene conversion) and *TbrPDEB2b* (converted allele). Interestingly, the converted stretch covers precisely the GAF-A domain, so that the gene conversion effectively resulted in the replacement of the entire GAF-A domain of TbrPDEB2 by its homologue from TbrPDEB1. The mechanism that converted *TbrPDEB2* is likely to be gene conversion since the length of the converted tract is short, between 297 and 600 bp (minimal and maximal converted tracts, respectively), and non-reciprocal. The size of the converted tract is considerably larger than the length of the minimal efficient processing segment determined for *T. brucei*, and well within the range determined for *Leishmania*, *S. cerevisiae* or mammals [19]. It is not possible to distinguish if *TbrPDEB2* recombined with the upstream allele of *TbrPDEB1* on the same or on the homologous chromosome. Also, it is not possible to decide if the altered allele *TbrPDEB2b* is the result of meiotic or mitotic gene conversion. A mitotic event should probably be favoured in view of the fact that meiosis still has not been demonstrated in *T. brucei* [36], and might occur very rarely, if at all.

Earlier studies from this laboratory had shown that, in the strain Lister427, the enzymes TbrPDEB1 and TbrPDEB2 exhibit different subcellular localizations. TbrPDEB1 is predominantly located in the flagellum, where it remains tightly associated with a detergent-resistant structure. In contrast, TbrPDEB2 is partly located in the flagellum and partly in the cell body as a soluble enzyme [3]. Studies with human PDEs [8], as well as data from our own laboratory with *T. brucei* (Luginbuehl et al., unpublished), indicate that the N-terminal regions of GAF-containing PDEs are involved in intracellular localization. The observation that in the 427 strain used for these localization studies [3], the GAF-A domain of one allele of TbrPDEB2 had be replaced by the GAF-A domain of TbrPDEB1 raised the question if the observed subcellular localization of TbrPDEB2 might have been influenced by this gene conversion. To clarify this, one allele of TbrPDEB2 was C-terminally tagged in procyclic forms of strain STIB247, a strain that has not undergone gene conversion. The subcellular localization of TbrPDEB2 in this strain was closely similar to that seen in the Lister427 strain. In conjunction, these data demonstrate that replacement of the GAF-A domain of TbrPDEB2b through gene conversion does not alter the intracellular localization of the enzyme. They indicate that the GAF-A domain may not contain the crucial signals for intracellular targeting.

To explore the presence of the gene conversion in a number of trypanosome strains, suitable primer pairs were developed for an easy PCR characterization of strains. PCR reactions using individual primer pairs allow an easy detection of the two alleles. A duplex PCR assay using a combination of three primers simultaneously detects TbrPDEB1 (as a positive control) and the TbrPBEB2b allele. Both approaches were applied to genomic DNAs of a variety of strains with different histories. The unambiguous outcome of these experiments showed that the gene conversion is only present in the Lister427 strain and all its derivatives, but that it is absent from all other independent isolates tested, including a *T. b. rhodesiense* strain. This finding makes detection of the gene conversion in TbrPDEB2b a convenient and reliable marker for identifying trypanosome strains, separating Lister427 derivatives from all others. While this may not represent a diagnostic tool of immediate value for field work, the technique is very useful in the laboratory environment whenever a potential mixup of strains or strain origins are an issue. An example is given by the continuous debate if the strain designated s427 is in fact the origin of the Lister427 derivatives (trypanosome pedigree section at http://tryps.rockefeller.edu/trypsru2_pedigrees.html). An other clear example for the potential usefulness of our analysis is presented in reference [37] (Peacock et al.): The observation that a derivative of Lister427 can be transmitted through the fly violates the dogma that this is a monomorphic strain and not fly-transmissible. This immediately begs the question if their Lister427 variant 3 is really a Lister427 derivative. Our analysis has now made this unambiguously clear.

This study has demonstrated the occurrence of gene conversion between two closely related and closely positioned PDE genes in *T. brucei*. The length of the converted fragment corresponds to those found in human gene conversions, and it corresponds precisely to the open reading frame of one protein domain, GAF-A. The conversion was found in only one trypanosome strain, Lister427. All its derivatives that have been produced over the last 40 years stably maintain this conversion in the heterozygous state. The presence of the converted gene does not alter the intracellular localization of the gene product, TbrPDEB2. In terms of the genetic mechanisms operating in *T. brucei*, the identification of a gene conversion event in a gene other than the much studied variant surface glycoprotein genes may provide further clues to the inner workings of the *T. brucei* DNA repair and recombination machinery.
